# Nursing Students’ Understandings of Mental Health: Illuminating the Structure of Awareness Using Phenomenographic Analysis

**DOI:** 10.1177/01939459261476388

**Published:** 2026-09-05

**Authors:** Sinead Barry, Louise Ward, Ruby Walter

**Affiliations:** 1 School of Health & Biomedical Sciences, STEM College, RMIT University, Bundoora, VIC, Australia; 2 Southern Cross University, East Lismore, NSW, Australia

**Keywords:** phenomenography, structure of awareness, qualitative research, nursing education, mental health

## Abstract

**Background:**

Variation in how nursing students conceptualise mental health (MH) has significant implications for curriculum design and patient care. While phenomenography is well-established for mapping such variation, clarity on how variations are derived is often lacking. Marton and Booth’s (1997) structure of awareness (SoA) framework provides a method for sophisticated analysis of a concept and, in this study, was employed to derive the components and configuration of students’ awareness.

**Objective:**

This study aimed to illustrate and advance the application of the SoA framework in phenomenographic analysis, uncovering both the range and the formation of undergraduate nursing students’ understandings of MH.

**Methods:**

A qualitative, phenomenographic design was employed. Nineteen diverse undergraduate nursing students from an Australian Bachelor of Nursing program participated in individual, semi-structured interviews. Data were analysed using Dahlgren and Fallsberg’s 7-phase process, with systematic application of Martin and Booth’s SoA framework to each emergent category.

**Results:**

Seven hierarchical categories of description were identified, representing a progression from fear-based and stigma-based views to holistic, inclusive conceptions of MH. Each category’s SoA foregrounded different aspects of MH, reflecting influences such as cultural context, personal experience, and educational exposure.

**Conclusions:**

Applying the SoA framework enriched phenomenographic analysis by revealing not only what students understood about MH but how their awareness was constructed and enacted. The insights assisted in the development of targeted strategies to enhance MH literacy, reduce stigma, and support the development of holistic, person-centred care in nursing education.

## Introduction

Mental health (MH) is a major component of contemporary nursing practice, with nurses frequently providing frontline care to people experiencing mental distress, MH crises, or co-occurring physical and MH conditions.^1,2^ Despite this, nursing students commonly report uncertainty, fear, and stigma when engaging with MH^2,3^ and variable exposure to MH content in curricula.^2^ MH literacy among nursing students is therefore critical, as their conceptualisations of MH can shape help-seeking, therapeutic relationships, and the quality of care provided.^2^ Understanding how nursing students conceptualise MH—what they bring into focus, what they background, and how they position themselves in relation to people experiencing MH issues—is important for informing educational interventions that foster safe, equitable, and person-centred care.

Variation in individuals’ experiences, conceptions, and understandings of phenomena is fundamental to phenomenography—a qualitative research approach that “map[s] qualitatively different ways people experience, conceptualize, perceive, and understand various aspects of, and the phenomena in, the world around them.”^4^^(p. 143)^ Within nursing education, identifying variation in how key concepts are understood is vital, as these differences have significant implications for curriculum design, student support, and patient care.^5,6^

Phenomenography, rooted in a non-dualistic, relational epistemology,^4,5^ seeks to capture the diverse ways learners make sense of their professional world. Within MH education, this approach allows researchers to examine how nursing students’ understandings of MH are shaped by, and in turn shape, their learning and practice environments.

Marton and Booth’s^7^ *structure of awareness* (SoA) framework offers an analytical lens recognising that a person cannot be aware of all aspects of a conception at the same time” and that “people can be aware of the same concept at the same time but in differing ways.”^8^ This framework delineates the referential and structural aspects of awareness—including internal and external horizons, the act, and the indirect object—across both “what” and “how” dimensions of understanding. Adopting this SoA moves analysis beyond categorisation enabling exploration of the configuration of awareness underpinning each way of knowing.^9,10^ In this study, “deeper” refers to making explicit how different ways of understanding are constituted in awareness—what aspects of MH are foregrounded or backgrounded (structural horizons), how students relate to or act towards the phenomenon (act and indirect object), and the meanings they attribute (referential aspect).

Applied in nursing education research, this analytical approach allows educators and researchers to “unpack” how MH is foregrounded or backgrounded within student awareness, shaped by cultural context, personal experience, and the biomedical model dominant in curricula.^11^ This understanding exposes barriers and facilitators to MH literacy—critical for designing interventions that move students towards more holistic, inclusive conceptualisations.^12^

Despite this potential, there remains limited methodological clarity regarding how variations in learners’ conceptions are analytically derived and articulated in phenomenographic research. This study addresses this gap by illustrating the application of Marton and Booth’s SoA framework to explicate undergraduate nursing students’ understandings of MH, thereby offering a transparent and theoretically grounded approach to deriving variation in conceptions.

## Objective

The objective of this study was to explore the different ways undergraduate nursing students understand MH and how these understandings are configured in awareness. We deliberately used the broad term MH and invited students to describe what it meant to them. Although the term was left open, students’ accounts predominantly orientated towards poor MH and MH disorders, particularly in relation to consumers and people in their personal lives; the findings therefore reflect variation in these understandings. To do so, we undertook a phenomenographic analysis incorporating Marton and Booth’s SoA framework,^7^ with a specific emphasis on illustrating how this framework can advance the analytic description of variation in students’ MH conceptions. The referential aspect captures the meaning of MH in a particular way of understanding; the structural aspect captures how this meaning is organised in awareness, as illustrated in Figure 1.

**Figure 1.**
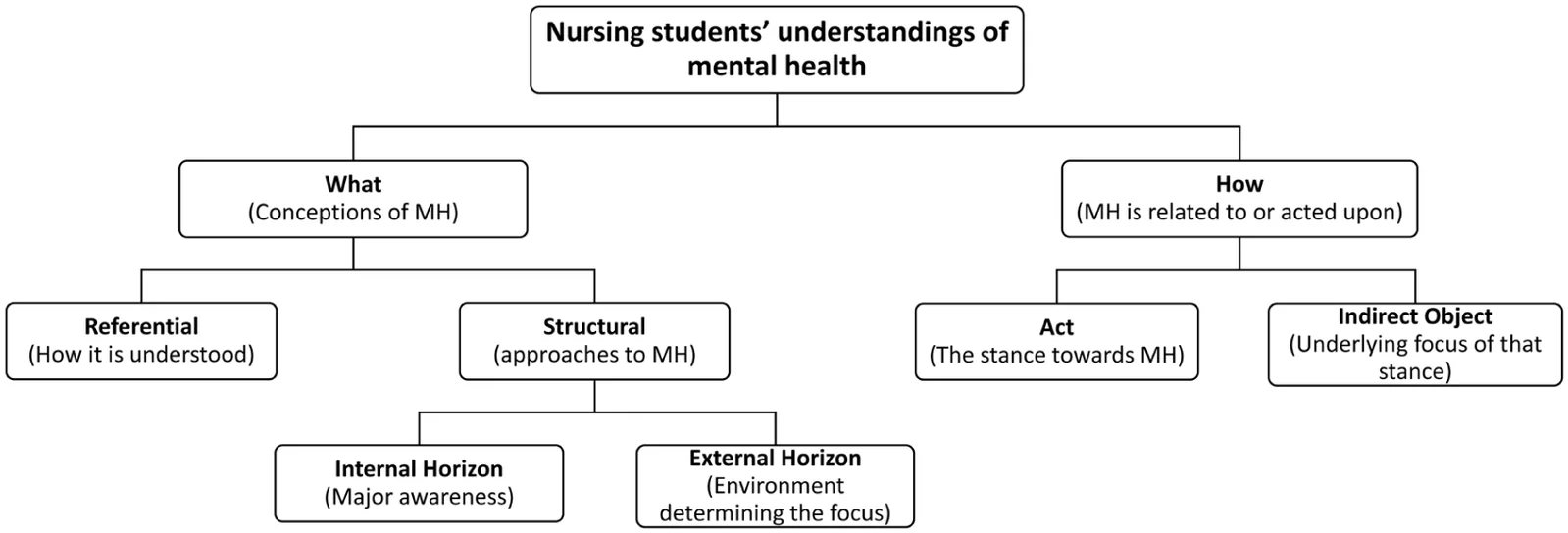
Structure of awareness of a way of understanding MH. MH, mental health. Adapted from Marton and Booth.^7^(p. 91)

## Methods

### Design

This qualitative study employed a phenomenographic design to investigate the different ways undergraduate nursing students understand MH. Phenomenography, recognised for mapping variation in learners’ experiences and conceptions,^5,7^ was chosen for its alignment with the research aim. The methodological innovation of this study centred on Marton and Booth’s SoA framework,^7^ which was used to analyse the “what” (referential and structural aspects) and “how” (act and indirect object) dimensions of students’ awareness.

### Participants

Nineteen undergraduate nursing students were recruited from a metropolitan Australian university’s bachelor of nursing (BN) programme. Purposive and snowball sampling aimed to recruit students with varied genders, cultural backgrounds, modes of entry, prior MH experience, and study pathways. Recruitment was facilitated through group e-mail invitations, study information at lectures, and participant referrals.

### Data Collection

Individual, semi-structured interviews were conducted to encourage reflection and elicit in-depth accounts of each student’s understanding of MH. The interview schedule comprised open-ended questions and targeted prompts. Interviews were conducted in a private setting on campus or at an affiliated clinical location, audio-recorded with consent, and transcribed verbatim.

In the interviews, we deliberately used the broad term MH without further definition and invited students to describe what it meant to them. This wording allowed participants to foreground aspects they considered most salient, whether MH as well-being, poor MH, or MH disorders. Table 1 illustrates the 2 core questions and 5 probing questions each participant was asked.

**Table 1.** Phenomenographic Interview Questions.

Interview questions
• What do you think mental health is? ○ Is there an experience that stood out and influenced your opinion? ○ Is there anything outside university that perhaps shaped your opinion of what mental health means to you?
• Thinking back on your time at university as an undergraduate nursing student how do you think your views of mental health have changed? ○ What was your views when you started and how has it changed? ○ On your first day of nursing, what did you think about mental health? ○ How has your understanding of mental health shifted since your first day of nursing?

### Data Analysis

Interview transcripts were the sole focus of the analysis. The first author inductively immersed to gain familiarity with the participants’ narratives. The analytic strategy followed Dahlgren and Fallsberg’s 7-phase phenomenographic process.^13^ Initial categories of description (CoD) and a draft outcome space were developed in hierarchical order.^14^ Once a stable set of categories was agreed upon, Marton and Booth’s SoA^7^ framework was applied as a second analytic step. For each category, we specified the referential aspect, structural horizons, and how-aspect (act and indirect object), to examine category coherence and distinctiveness. In this way, SoA operated as a systematic interpretive layer that made explicit the analytic rationale for the categories and their hierarchy. Figure 2 summarises the key analytical stages.

**Figure 2.**
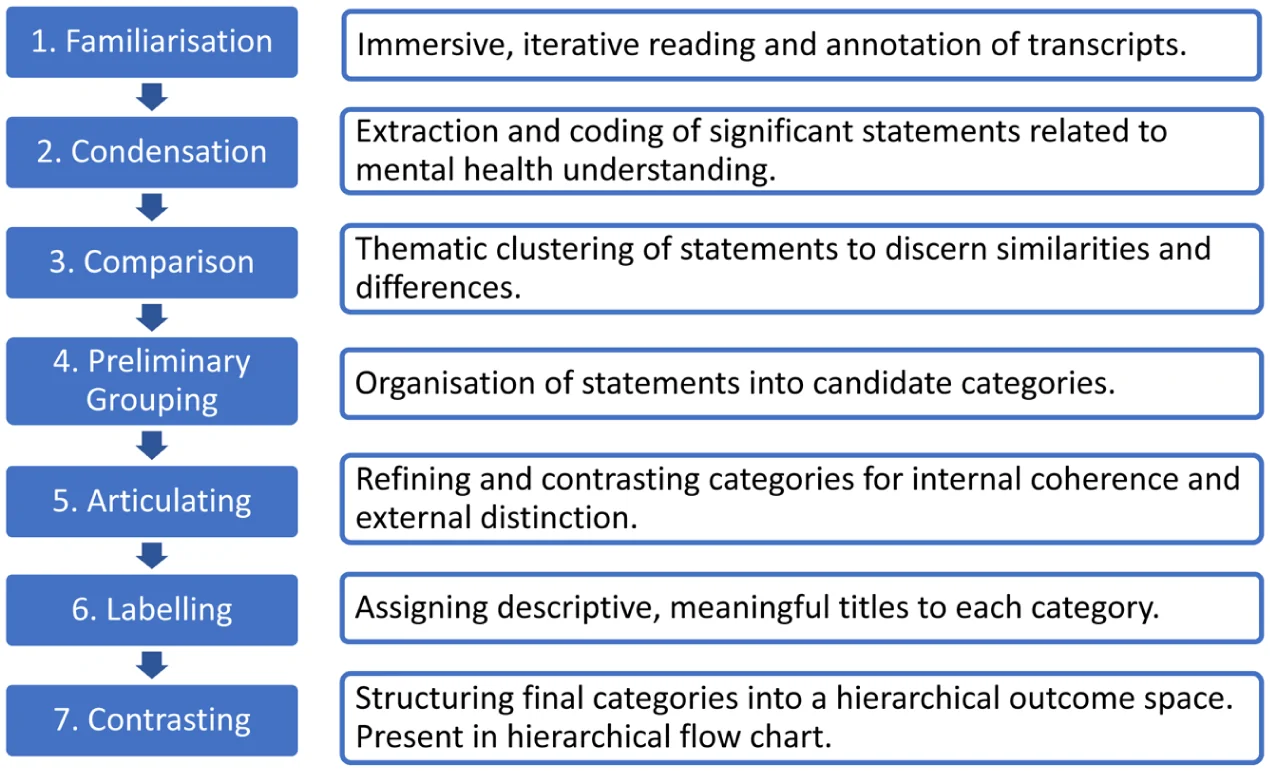
Phenomenographic process of data analysis.

Throughout analysis, the SoA was applied to each CoD. *Referential aspect* (the meaning attributed), *structural aspect* (internal/foregrounded and external/contextual horizons), and *how-aspect* (mode or act of understanding, indirect object/intention), revealed the underlying configuration of participants’ awareness. In conventional phenomenographic reporting, analytical work typically culminates in the identification of CoD and their organisation into an outcome space. While this captures what qualitatively different ways of understanding exist and how they relate, the basis for distinguishing categories may remain implicit. By systematically applying the SoA framework within each category, we made explicit the analytic moves underpinning category formation clarifying why categories differ and revealing intra-category variation and the dynamic configuration of awareness.

Rigour was enhanced through ongoing reflexive and collaborative practices, informed by contemporary guidance on reflexive qualitative practice.^15^ The first author, a lecturer in nursing and MH nurse, kept a reflexive journal to document prior assumptions, emotional responses, and emerging interpretations; these notes were revisited to check whether readings might reflect researcher stance rather than participants’ accounts, prompting return to the data where needed. Analytic meetings with co-authors, who held differing relationships to nursing, MH, and phenomenography, were used to review transcripts, SoA matrices, and emerging categories. Disagreements were recorded in analytic memos and resolved through collective re-reading and refinement of categories. External feedback from a phenomenographic expert and a senior academic in nursing informed the labelling and description of categories, supporting transparency and credibility.

### Ethical Considerations

Ethics approval was obtained from the institutional Human Research Ethics Committee (application no 15-098) based on the sensitivity of the participant profile and the topic of MH. Participants provided written informed consent after receiving information about the study, risks, and confidentiality. Protocols were in place for managing participant distress, including immediate referral to university counselling and support services. The dual role of the researcher (PhD candidate and nurse educator) was disclosed to all participants, with voluntary participation and withdrawal rights explicit. Data were de-identified, securely stored, and only accessible to authorised personnel, maintaining strict privacy throughout.

## Results

Nineteen undergraduate nursing students participated in the study. Participants were enrolled in the BN or bachelor of nursing and midwifery (BNBM) across first, second, and third year at a metropolitan Australian university. Most participants were women (79%; men 21%) and Australian nationals (68%; 32% from other countries). The majority were enrolled in the BN (95%; BNBM 5%), with representation across first (21%), second (32%), and third year (47%) of study. Most identified as mature-age or post-school entrants (84%); only 16% enrolled in university immediately after completing secondary school.

A total of 7 CoD representing the variation in nursing students’ understandings of MH are presented in Figure 3. Higher order categories progressively incorporate and reorganise these earlier elements, broadening what is foregrounded and shifting the stance from distance and “othering” towards lived experience and holistic well-being. The outcome space visually represents the ways the students understood MH.

**Figure 3.**
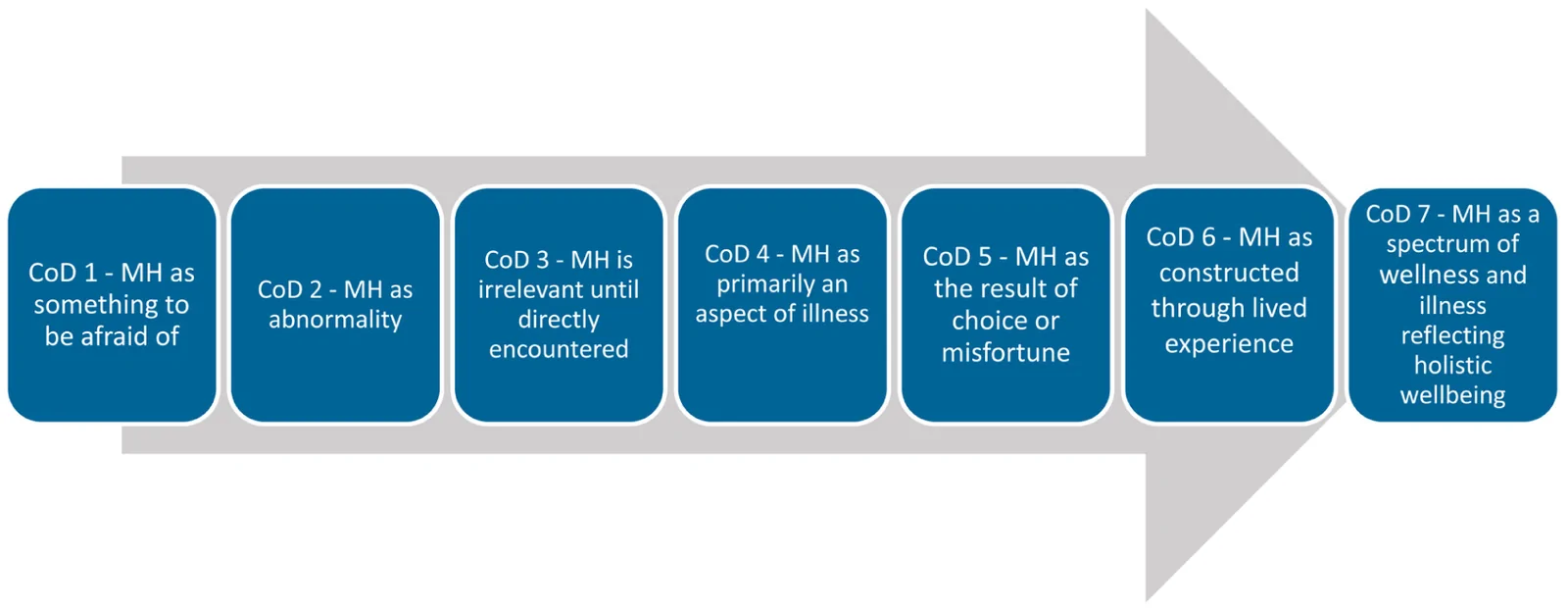
CoD representing undergraduate nursing students’ understandings of MH. CoD, categories of description; MH, mental health.

Figure 4 visually represents the components of the students’ awareness for each of the 7 CoD. Each of the 5 branches shown in Figure 4—referential, internal horizon, external horizon, act, and indirect object—are explained. Following each CoD across the horizontal line illustrates the “what” and “how” factors influencing students’ understandings.

**Figure 4.**
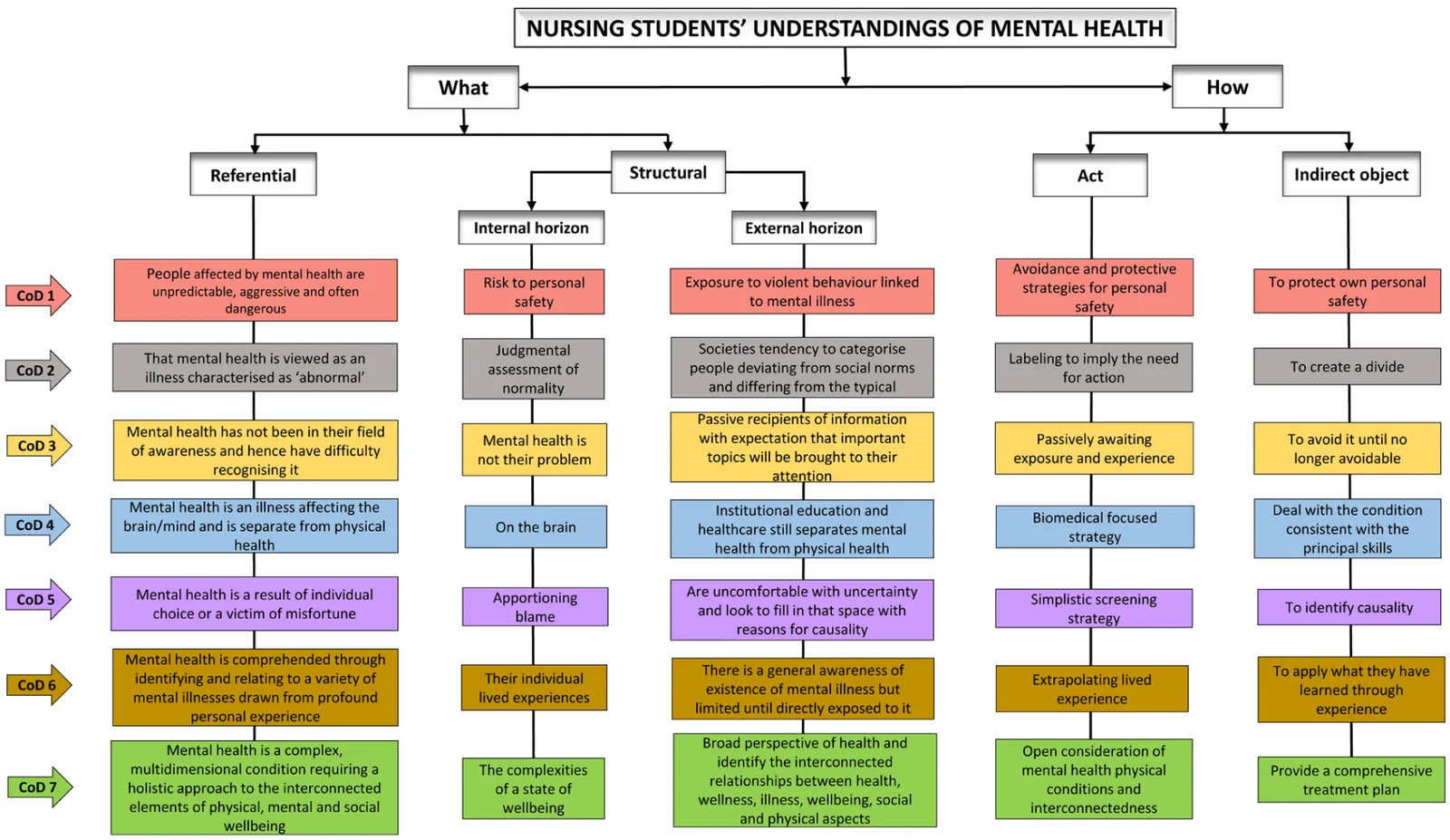
Structure of awareness illustrating nursing students’ understandings of mental health.

Figure 4 also illustrates how the SoA analysis extends beyond naming categories. For each way of understanding MH, the figure shows not only the referential meaning but also which aspects of MH are central in awareness (internal horizon), which remain peripheral or background (external horizon), and the kind of relationship or stance students take towards MH (act and indirect object). These patterns clarify how one way of understanding differs from another—for example, shifts from individual blame to contextual sensitivity, or from symptom-focussed noticing to relational engagement—which would be less visible if we only reported the categories and their hierarchical arrangement.

### Category 1: MH as Something to Be Afraid of

In this way of understanding, MH is configured primarily as a source of danger. Students foregrounded the perceived unpredictability and aggression of people experiencing MH issues, backgrounding contextual, relational, or recovery-orientated aspects of their lives. This configuration positioned students in a defensive stance, with the central aim of protecting themselves and others. Students’ talk revealed a referential focus on MH as severe mental illness and violence, as illustrated by one participant:

When I was quite young it was quite scary ‘cause I had one friend whose brother was severely mentally ill and aggressive and stuff, so that probably was quite scary. . . He was really unwell and undiagnosed. . . and quite violent as well. (Participant 10)

Within the internal horizon, personal safety was foreground, orientating students’ action towards vigilance and self-protection:

I would try. . . be on the back foot. . . to make sure I am not injured, or if he turns aggressive towards me. . . just try to take the aggression off. (Participant 14)

The external horizon reflected cultural messages that reinforced fear and avoidance, such as advice to distance oneself and leave management to authority figures:

They would probably tell everyone to stay away from that person and only the leaders/elders can deal with that person, and sometimes they will restrain them. . . cuff their hands and stuff like that. (Participant 8)

Consequently, the dominant act involved cautious engagement or avoidance—approaching MH encounters as situations to be managed carefully to prevent escalation:

You have just got to be cautious in how you approach them ‘cause you don’t want to have someone spike them. . . I just like to be very cautious in mental health areas. (Participant 14)

Across these accounts, the indirect object of students’ awareness was self-protection. However, some students noted that increased exposure during placement began to disrupt this fear-dominated configuration:

Other people would be scared. . . they will hit us, they will harm us, hurt us. So these were some of the fears in my mind. . . but when I went on placement, they all disappeared. (Participant 9)

### Category 2: MH as Abnormality

In this way of understanding, MH is configured as a deviation from what is considered “normal.” Students foregrounded difference, dysfunction, and loss of control, while backgrounding contextual and relational dimensions of people’s lives. This awareness structure positioned people experiencing MH issues as “other” and reinforced a boundary between “normal” and “abnormal” that underpinned stigmatising attitudes.

The referential aspect centred on MH as abnormality or illness, marked by behaviours that departs from taken-for-granted norms:

Mental health to me is being abnormal, not being able to cope with the functions of normal life—I suppose an illness. (Participant 16)

Within the internal horizon, students’ understandings were shaped by deep-seated societal labelling in which “mental” was used as a derogatory term and closely tied to illness and inferiority:

In the place where I was brought up. . . there were people who used to treat mental health as a stigma; like people who were mentally ill, they used to treat them badly, and they used the word ‘mental’ for negative use only. . . it had some impact on me like mental means illness or something like that. (Participant 17)

The external horizon highlighted broader societal and cultural norms that define what counts as health or acceptable ways of thinking and behaving, and how these judgements vary across contexts:

I think mental health is much broader and individualised, and I think it also has got to do with culture—like in some cultures the way some people think might not be considered healthy in other cultures. (Participant 10)

Within this configuration, labelling functioned as an act that implied the need for action or correction, drawing a clear line between those deemed “normal” and those with MH issues:

A really ‘normal’ [person] would probably be someone who doesn’t have mental health illness. (Participant 15)

The indirect object of students’ awareness was the maintenance of this “normal/abnormal” divide, reinforcing “them and us” thinking. However, some accounts also pointed to the possibility of this divide being challenged when students encountered people with lived experience who did not fit the stereotype:

Well, at first, I thought people would be having really terrible side effects. . . it wouldn’t be normal human beings, or normal citizens contributing to society. But after my mental health placement. . . some of those people were actually contributing to society and holding down stable jobs. (Participant 15)

### Category 3: MH Is Irrelevant Until Directly Encountered

In this way of understanding, MH is configured as distinct and largely irrelevant to students’ lives. MH concerns are foregrounded only when directly encountered, while everyday relevance, ongoing societal impact, and personal responsibility remain backgrounded. This awareness structure positions MH as “someone else’s issue” that does not demand attention until it intrudes into students’ immediate experience.

The referential aspect revealed MH as something that exists primarily outside students’ personal worlds, often linked to a lack of prior exposure:

I grew up in a rural town, so that is number one. . . for not having had any contact with anyone who had a mental illness and had sought help. (Participant 5)

Within the internal horizon, students described being unaware of what MH issues might feel like or entail, placing MH outside their own concern or imagination:

It started off as. . . well, I was unaware of what it would feel like or be like. I didn’t know, I wouldn’t know. (Participant 16)

The external horizon showed that MH remained largely invisible in students’ families and communities unless explicitly addressed, with responsibility often deferred to others:

It was never spoken about, to be honest. It was left to the elderly to handle that. It was not left to the younger generation. (Participant 8)

Consistent with this configuration, the dominant act involved passive waiting rather than active engagement, with MH only becoming salient when raised in formal settings or through direct encounters:

We have not touched on mental health whatsoever in uni—we haven’t even spoken about it. (Participant 12)

The indirect object was the avoidance of engagement until contact with MH issues became unavoidable, particularly for those from context where such issues were rarely visible of openly discussed: “I grew up in a rural town. . . no contact with anyone who had a mental illness” (Participant 5).

### Category 4: MH as Primarily an Aspect of Illness

In this way of understanding, MH is configured primarily as an illness located in the brain. Students foregrounded biological dysfunction and diagnostic categories, while backgrounding social, relational, and experiential dimensions. This awareness structure positioned MH as a medical condition to be treated, rather than as a broader aspect of well-being.

The referential aspect centred on MH as a physical or neurobiological condition, often linked to genetics, brain injury, or chemical imbalance:

I think mental health is a condition. . . usually in the brain and could have developed from things people had experienced or be a genetic condition they were born with. (Participant 1)

Within the internal horizon, students’ attention was directed towards the brain and cognitive functioning, emphasising mental abilities as the core locus of MH:

Mental health refers to the brain and cognitive ability of an individual. . . what mental health means to me is how can we deal with our mental abilities. (Participant 17)

The external horizon reflected a perceived separation between mental and physical health, reinforced health care structures that treat them as distinct domains rather than aspects of a whole person:

. . . the idea it’s still completely separate from physical health. . . so people often don’t look at the fact that their patient who is here [is a whole person]. (Participant 3)

Within this configuration, the dominant act involved applying a biomedical lens—understanding MH in terms of thinking, feeling, and diagnosis, and orientating towards clinical management:

Mental health is just the mind and thinking and feeling healthy, I think. (Participant 10)

The indirect object was to deal with MH as an illness of the mind, reinforcing a disease-based framing:

Mental health is a disease of the mind. (Participant 2)

### Category 5: MH as the Result of Choice or Misfortune

In this way of understanding, MH is configured as the consequence of a specific trigger—either an individual’s choices or unfortunate life events. Students foregrounded causation and personal narratives of “what went wrong,” while backgrounding more complex, multifactorial accounts of MH. This awareness orientated students towards searching for causes and, at times, allocating blame, even as some recognised contextual factors and move towards reduced judgement.

The referential aspect centred on MH as something that does not “just happen,” but brought about by actions, circumstances, or moral trajectories:

I used to have the thought that mental health was always self-inflicted somewhere along the line; it didn’t just happen. It’s been caused by someone’s actions, whether by them or those around them. (Participant 4)

Within the internal horizon, students described an active search for the turning point or cause, a curiosity that could lead to locating fault:

Finding out their story and knowing, well, where did their paths cross and change, so to speak. I think that will always make me curious—like where did something go wrong. . . to then snowball into a mental illness. (Participant 4)

The external horizon reflected discomfort with uncertainty and a need for explanation, including attempts to normalise emotional response to adverse events:

. . .you might get people that have cancer with depression; I mean it’s a normal sort of response. (Participant 1)

Within this configuration, the dominant act involved screening for causes—initially in ways that risked not only oversimplifying or individualising responsibility but also opening space to recognise broader contributing factors:

Anybody could become homeless or turn to substances to make themselves feel better; it’s not by choice. (Participant 4)

The indirect object was the identification of causality, which, for some students, supported a shift towards less judgemental understandings as they recognised underlying problems and contextual influences:

. . .definitely less judgemental; I no longer say, ‘Oh, how did they ever do that?’, ‘Why would they ever get into drugs?’. . . I have noticed underlying problems that may have made them do that to start off with. (Participant 8)

### Category 6: MH as Constructed Through Lived Experience

In this way of understanding, MH is configured through personal and relational experiences rather than through abstract concepts. Students foregrounded their own and others’ lived experiences as primary sources of insight, while backgrounding purely theoretical or second-hand accounts. This awareness structure fostered humility, openness to learning, and greater empathy towards people living with MH issues.

The referential aspect centred on MH as something understood progressively through experience, with students recognising both the value and limits of their current knowledge:

I am thankful I have had experiences like that. But then. . . what I know. . . would only scrape the surface; there is still so, so much to learn. (Participant 12)

Within the internal horizon, students described how close relationships with people experiencing mental illness reshaped their understandings in way that surpassed formal learning or media portrayals:

I have had a family member. . . and friends who have been through mental illness. . . it is even more of an insight. . . than watching a documentary, or whatever, as I know that person so closely and know what they have been through. (Participant 7)

The external horizon involved recognition that, without direct exposure, MH had previously carried negative connotations or stereotypes and that encountering lived experience could disrupt these assumptions:

Mental health used to have a bit of a negative connotation for me, but now since I have actually experienced it. . . you can function quite well having a mental health issue. . . (Participant 13)

Within this configuration, the dominant act was extrapolating from lived experience—using changes in one’s own or close other’s MH to deepen understanding of what it is life to live with such challenges:

And then. . . my own mental health has changed a bit so I have gained a greater understanding of what it’s like to experience that. . . (Participant 3)

The indirect object was the application of learning from these experiences to broader views of MH, drawing on a constellation of personal, family, and professional encounters:

. . . it is a collection of all my experience with it, I guess. . . my grandmother had dementia. A close family friend is a psychiatrist. . . people I have known have committed suicide, so that has probably influenced my views on mental health. . . (Participant 10)

### Category 7: MH as a Spectrum of Wellness and Illness Reflecting Holistic Well-Being

In this most inclusive way of understanding, MH is configured as a multidimensional state of well-being that integrates physical, social, emotional, and psychological aspects of life. Students foregrounded interconnectedness and continuity (rather than illness alone), while backgrounding rigid separations between “mental” and “physical” health. This awareness structure positioned MH as both illness and wellness, embedded in everyday living and central to person-centred care.

The referential aspect centred on MH as a broad, dynamic spectrum, encompassing illness, wellness, and all points in between:

Mental health is our internal state. . . can be characterised by mental illness or wellness, or it’s sort of a spectrum in between. (Participant 3)

Within the internal horizon, students acknowledged MH as complex and woven into “normal” life, even illness is present, rather than something separate or exceptional:

. . . if you have a mental illness, you have to go to work and may do further study, or whatever you have got to do to raise a family. . . mental health stuff just comes along with that. . . (Participant 3)

The external horizon highlighted a broad sense of interconnectedness, with MH understood as inseparable from other dimensions of health and well-being:

. . .it can come under pretty much your physical, social and emotional health. There is not really a definitive line for me. . . (Participant 14)

Within this configuration, the dominant act involved open-minded integration and a person-centred stance, recognising that mental well-being influences—and is influenced by—all aspects of life:

For me you need to have good mental health in order to have good physical health. I think your mental wellbeing affects all aspects of your life. . . (Participant 2)

The indirect object was comprehensive treatment and broad well-being, with students orienting towards understanding and responding to the full scope of a person’s psychological and contextual circumstances, rather than focussing solely on pathology:

I guess it would cover everything to do with people’s psychological wellbeing, their psychiatric wellbeing; anytime someone is not feeling good. . . there is a negative impact on their psychological wellbeing—sometimes it’s an environmental thing, other times it is pathological. (Participant 11)

The hierarchical ordering reflects increasing complexity and inclusiveness of students’ awareness rather than a simple good-bad distinction. Earlier conceptions are understood as developmentally and contextually shaped starting points, which can coexist with, or be transformed into, more expanded ways of understanding.

## Discussion

The application of Marton and Booth’s SoA^7^ framework provided a nuanced lens to understand how nursing students conceptualise MH, going beyond what they know to consider how their awareness is constructed, prioritised, and enacted. This approach highlighted that nursing students’ understanding of MH is not static but shaped by the dynamic interplay of referential and structural aspects, internal and external horizons, and intentional actions.^9,16^ The analysis confirmed wide variation among students—from understandings rooted in fear, stigma, and “othering,” to those incorporating more holistic, inclusive, and wellness-oriented perspectives.^2,12^

The findings suggest that attending to the SoA can make visible how particular facets of MH are foregrounded or backgrounded, helping to explain differing conceptions and the barriers that may impede the development of MH literacy. Many students, for example, foregrounded narratives of fear or abnormality, shaped by media, cultural beliefs, and institutional discourses and formal curriculum.^16-18^ In these cases, the indirect object of awareness often centred on self-protection or the maintenance of boundaries, while the act revolved around avoidance or labelling—mechanisms that perpetuate stigma and restrict empathetic engagement.^19-21^

Conversely, students whose SoA included lived experience—either their own or that of close contacts—showed a marked shift in focus. Here, the internal horizon broadened, accommodating greater nuance, reflective capacity, and positive attitudes and empathy.^22,23^ The act transitioned from avoidance or judgement to reflection and application, consistent with wider literature that points to the potential value of contact-based learning and narrative approaches as anti-stigma strategies in nursing education.^12,24^ The highest order understandings encapsulate the interconnectedness of mental, physical, emotional, and social health, aligning with contemporary holistic models and World Health Organization definitions,^25^ and these students articulate intentions consistent with person-centred, inclusive practice.^2^

From a methodological perspective, the main contribution of this study lies in illustrating how the SoA framework can be used to illuminate these shifts and tensions. Rather than only identifying CoD and an outcome space, the analysis made explicit how stigma and compartmentalisation, as well as more integrated conceptions, are configured in awareness. Educational implications should be interpreted as illustrative. The findings indicate that educational strategies might be conceptualised as supporting students to build on, and critically reflect on, their existing understandings, rather than simply replacing “incorrect” conceptions with “correct” ones. Curriculum and pedagogy could, for example, be mapped to target both what is present in students’ awareness and what remains background.^5,6^ By surfacing the intentional dimensions and contextual influences implicit in students’ thinking, educators may be better positioned to support developmental progression from simple, dichotomous understandings towards more flexible, integrated, and compassionate conceptions.^26^

To illustrate, students in the early categories foreground danger, difference, and self-protection, while contextual factors, lived experience, and recovery remain backgrounded, and the dominant act is avoidance or labelling. A curriculum that takes this configuration seriously might incorporate structured contact-based learning activities in which students hear from people with lived experience of MH disorders,^24^ combined with guided reflection that explicitly invites students to notice shifts in what they foreground and how they position themselves.

Similarly, for students whose awareness is configured around MH as primarily an illness of the brain, case-based learning could be designed to juxtapose biomedical information with narratives highlighting social determinants, trauma, and strengths. Facilitated discussions could prompt students to identify what they foreground or background and to experiment with alternative acts (relational engagement, advocacy) and indirect objects (holistic well-being, recovery).

Methodologically, the integration of SoA was closely intertwined with our reflexive practices. The first author’s background in MH nursing, and the team’s differing proximities to clinical practice and phenomenographic research, periodically led us to privilege certain meanings in early readings. Reflexive journaling and collaborative review helped us identify these tendencies and re-examine transcripts. This process underlines that the SoA was not a neutral technical tool, but one shaped by and used to critically interrogate our own positions in relation to nursing, MH, and the students’ accounts. Rather than claiming statistical generalisability, our intention is to offer a theoretically informed account of variation in students’ understandings that may be transferable to contexts with similar educational structures and cultural influences.^27^

In summary, this study illustrates how using the SoA within a phenomenographic approach can provide insights for nursing education and research. It not only captures the diversity of students’ understandings but also offers one way of articulating the mechanisms by which those understandings are formed and may be transformed. While the study does not evaluate educational or clinical outcomes, the analysis points to how the SoA might inform the design of targeted curriculum strategies that recognise, respect, and respond to the deeply personal—and often hidden—aspects of learner’s professional development. Future work could examine whether using the SoA-informed approach influences learning and, ultimately, care that aspires to be inclusive, holistic, and responsive to the complexities of lived experience.

### Limitations

This study has several limitations that are important for interpreting its contribution and assessing its transferability.^27,28^ The findings are based on a single cohort of undergraduate nursing students in one Victorian University in Australia, and contextual influences on their understandings of MH may differ in other programmes or regions. As with all phenomenographic work, the CoD and outcome space represent the researcher’s interpretive constructions of variation, rather than an exhaustive taxonomy of students’ conceptions. The integration of the SoA framework, while offering analytic transparency, also reflects our theoretical commitments and may have foregrounded some features of the data over others. In addition, participants self-selected into the study, which may mean that students with strong views or greater interest in MH were over-represented. These limitations do not invalidate the findings, but they indicate that transferability should be considered in relation to similarities in context and that further work is needed to explore how SoA informed analyses play out in other educational and cultural settings.

## Conclusion

The 7 CoD illustrate a shift from fear, stigma, and superficiality towards lived experience and holistic, person-centred understanding. Direct experience, openness, and exposure to MH challenges are key in promoting more expanded and compassionate views. Embedding these findings within nursing education can help nurture MH literacy and reduce stigma among future nurses. The SoA framework enriches phenomenographic analysis by enabling researchers to dissect the nuanced layers of participants’ understanding—not only what differs but how these differences are constructed in awareness. This paper demonstrates its utility within nursing education research, offering a replicable analytical approach for future studies seeking to expose variations in complex conceptual domains.
